# Novel DERMA Fusion Technique for ECG Heartbeat Classification

**DOI:** 10.3390/life12060842

**Published:** 2022-06-06

**Authors:** Qurat-ul-ain Mastoi, Teh Ying Wah, Mazin Abed Mohammed, Uzair Iqbal, Seifedine Kadry, Arnab Majumdar, Orawit Thinnukool

**Affiliations:** 1 Faculty of Computer Science and Information Technology, University of Malaya, Kuala Lumpur 50603, Malaysia; quratulain.mastoi@siswa.um.edu.my (Q.-u.-a.M.); tehyw@um.edu.my (T.Y.W.); 2 College of Computer Science and Information Technology, University of Anbar, Ramadi 31001, Iraq; mazinalshujeary@uoanbar.edu.iq; 3 Department of Computer Science, National University of Computer and Emerging Sciences, Islamabad Chiniot-Faisalabad, Chiniot 35400, Pakistan; uzair.iqbal@nu.edu.pk; 4 Department of Applied Data Science, Noroff University College, NO-4608 Kristiansand, Norway; skadry@gmail.com; 5 Faculty of Engineering, Imperial College, London SW7 2AZ, UK; a.majumdar@imperial.ac.uk; 6 College of Arts, Media, and Technology, Chiang Mai University, Chiang Mai 50200, Thailand

**Keywords:** cardiovascular disease, ECG signal processing, features extraction, machine learning, ECG heartbeat classification

## Abstract

An electrocardiogram (ECG) consists of five types of different waveforms or characteristics (P, QRS, and T) that represent electrical activity within the heart. Identification of time intervals and morphological appearance of the waves are the major measuring instruments to detect cardiac abnormality from ECG signals. The focus of this study is to classify five different types of heartbeats, including premature ventricular contraction (PVC), left bundle branch block (LBBB), right bundle branch block (RBBB), PACE, and atrial premature contraction (APC), to identify the exact condition of the heart. Prior to the classification, extensive experiments on feature extraction were performed to identify the specific events from ECG signals, such as P, QRS complex, and T waves. This study proposed the fusion technique, dual event-related moving average (DERMA) with the fractional Fourier-transform algorithm (FrlFT) to identify the abnormal and normal morphological events of the ECG signals. The purpose of the DERMA fusion technique is to analyze certain areas of interest in ECG peaks to identify the desired location, whereas FrlFT analyzes the ECG waveform using a time-frequency plane. Furthermore, detected highest and lowest components of the ECG signal such as peaks, the time interval between the peaks, and other necessary parameters were utilized to develop an automatic model. In the last stage of the experiment, two supervised learning models, namely support vector machine and K-nearest neighbor, were trained to classify the cardiac condition from ECG signals. Moreover, two types of datasets were used in this experiment, specifically MIT-BIH Arrhythmia with 48 subjects and the newly disclosed Shaoxing and Ningbo People’s Hospital (SPNH) database, which contains over 10,000 patients. The performance of the experimental setup produced overwhelming results, which show around 99.99% accuracy, 99.96% sensitivity, and 99.9% specificity.

## 1. Introduction

Cardiovascular diseases (CVDs) are the leading cause of death globally, as reported by the World Health Organization [1]. Sustainable development has been made in past years for reducing the impact of heart morbidity. An electrocardiogram (ECG) signal is the most widely utilized biosignal for CVDs detection on an early basis. The ECG is a non-invasive measurement of the heart that is utilized to diagnose different cardiac illnesses and anomalies [2,3]. Cardio specialists have been utilizing ECG waveforms for over seven decades to distinguish heart illnesses, for example, arrhythmia and myocardial areas of dead tissue for more than 70 years [4].

Many cardiovascular diseases can be detected using the analysis of variation in ECG waves. However, the presence of artefacts such as baseline wandering and powerline interference in ECG signals can generate additional spikes in the waveform. Therefore, it is mandatory to eliminate the noisy elements from ECG signals to classify heartbeats accurately. The wavelet transforms technique is one of the most commonly used to remove noise and artifacts from ECG signals [5,6,7]. There have been several algorithms previously proposed to extract waveforms from the ECG cycle [8,9,10,11]. The authors in [12] proposed a rapid ramp effective algorithm to determine the occurrence of the R-peaks from ECG signals. This algorithm has higher-order complexity, and it is only applied to two records. Moreover, Hilbert transform and empirical mode decomposition are combined to detect the R-peaks from the ECG signal [13]. However, this approach is quite complex due to the consideration of massive amounts of blocks for detecting R-peaks. R peak detection is the sole purpose of both algorithms. In literature, some of the algorithms are proposed techniques to detect the P- and T-waves with R-peak [14,15]. These techniques include several steps, such as filtering the signals, creating interesting blocks for each peak individually and setting the fixed threshold point. To extract the large values from ECG signals, this algorithm specifically used the Butterworth filter to remove the baseline drift and square the output of the filtered signal. The selection of the area of the window depends on the length of the QRS complexes and repetition intervals. Each block’s width is computed based on the size of the window and compared with the threshold. Eventually, peaks are detected from each width of the window. The values of the prior and after of the detected R-peaks are selected and samples of the R-peaks are adjusted to 0 for the identification of P- and T-waves. As far as peak detection is concerned, this algorithm yields acceptable results. In this study, we propose a novel dual event-related moving average-based fusion technique, which allows extracting the specific events from the ECG signals, including P-, QRS complex, and T-waves. However, these specific events of the ECG signal contain time intervals due to the time frequency-based analyses that produce huge variations related to data from the specific waves. Furthermore, this paper demonstrates that the proposed technique significantly achieves better performance than the state-of-the-art methods. Other major aim of this study is to classify five types of heartbeats (PVC, LBBB, RBBB, PACE, and APC) from ECG signals to identify the exact condition of the heart. This aim includes two stages: to extract the features and classification using the supervised model. MIT-BIH arrhythmia benchmark dataset has been widely used for many similar experiments in the last decade [16]. Previous research employed a variety of preprocessing approaches, feature extraction methods, and classifiers, some of which are covered in this study. In [17], discrete-wavelet-transform (DWT) was used to extract R-peak and RR interval, whereas multi-layer perceptron (MLP) was utilized for classification. The accuracy was achieved at 99.9%, with 301 features in classification. Similarly, in [18], db4 DWT was used to extract the R-peak location and RR interval, support vector machine classifier was used to detect the abnormality from ECG signals. In [19,20], various classifiers were utilized for the classification of ECG signals, such as naïve Bayes, SVM, ANN, and support vector machines.

The novel proposed technique has the potential to be applied to identify the normal and abnormal events of the ECG signals. This type of system involves implanted probe-less ECG sensors on the patient’s body and transmitting the signals via Bluetooth connection to the processing device, for instance, a cellphone device, handheld device, and wearable wireless device. The obtained signal can be used to pre-process and extracts features for the classification of five heartbeat classes, namely PVC, LBBB, RBBB, PACE, and APC. The main highlights of the work are as follows:

1. We designed a fusion-based method DERMA with FrlFT to preprocess the ECG signal efficiently and extract the features such as P-, QRS, and T-waves.
2. Our proposed method specifically identifies the abnormal morphological events that are associated with PVC, LBBB, RBBB, PACE, and APC.
3. We calculated the temporal values, which are associated with specific events such as PR and RT waveform duration.
4. Furthermore, this study performed classification using supervised learning models, such as KNN and SVM, by utilizing the MIT-BIH Arrhythmia dataset, whereas two different datasets were utilized, namely AHA and SPNH, for testing purposes.

The organization of the paper is as follows, Section 2 discusses materials and methods, and Section 3 describes the classification of heartbeats. Section 4 discusses the results of the proposed model, and Section 4 provides the conclusion of this study.

## 2. Materials and Methods

**Figure 1:**
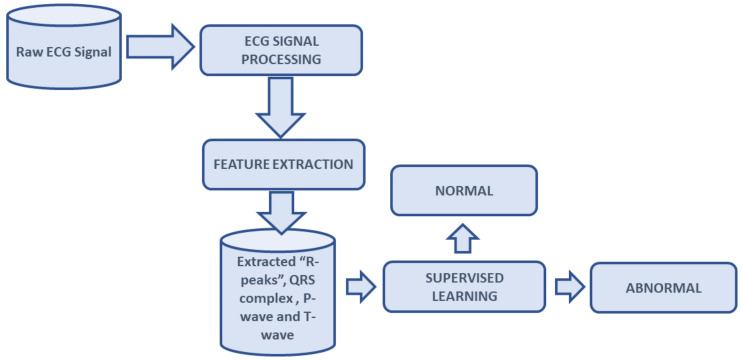
Block diagram of novel DERMA technique.

To detect the accurate waveform from ECG signals, it is mandatory to preprocess the ECG signals as discussed in the introduction. Figure 1 represents the block diagram of the proposed methodology, which includes the following steps: (1) to eliminate the noise from the ECG signal, (2) to detect the specific events from the ECG signals using DERMA with the FrlFT fusion technique, and (3) to select the supervised model for the classification of ECG signals for the determination of CVD. Each task of the proposed methodology is briefly discussed in the following subsections.

### 2.1. Preprocessing of ECG Signals

In this section, the first and foremost step is to normalize ECG signals to obtain the essential highest information. As we know, the presence of noise in ECG signals is non-linear and dependent on time whereas, amplitudes measurement is present in each subject, so it is necessary to calculate the statistical mean of the amplitude and then the difference among the $x\left(i\right)$ and μ by using the following equation:

(1) $$x\left(i\right)-\left(\mu \right)$$

μ is the statistical mean of the amplitude; σ is the standard deviation of the sample. The Z-score method is used to normalize all amplitude values from the ECG signals

(2) $$z=\frac{x\left(i\right)-\left(\mu \right)}{\sigma }$$

Discrete Wavelet Transform: determine the detailed co-efficient of ECG signals *x*(*i*) DWT of a function that is defined as follows [21]:

(3) $$W_{\phi }\left(S_{o},k\right)=\frac{1}{\sqrt{M}}\overset{M-1}{\underset{k-0}{\sum }}x\left(t\right)\phi_{S_{0},k}\left(t\right)$$

Additionally,

(4) $$W_{\psi }\left(S,k\right)=\frac{1}{\sqrt{M}}\overset{M-1}{\underset{k-0}{\sum }}x\left(t\right)\psi_{S,k}\left(t\right)$$

It is stated that $\geq S_{0}$, where $S_{0}$ represents starting scale, and $\phi_{S,k}\left(t\right)$ and $\psi_{S,k}\left(t\right)$ are defined as the scaling and wavelet function. The detailed coefficient for the inverse discrete wavelet transform is presented below:

(5) $$x\left(t\right)=\frac{1}{\sqrt{M}}\overset{s-1}{\underset{s_{0}=0}{\sum }}W_{\phi }\left(s_{o},k\right)\phi_{s_{0},k}\left(t\right)+\frac{1}{\sqrt{M}}\overset{s-1}{\underset{s=0}{\sum }}W_{\psi }\left(s_{o},k\right)\psi_{s,k}\left(t\right)$$

Fractional Fourier Transform (FrlFT): The FrlFT is a generalized variant of the conventional Fourier transform that includes a parameter α. FrlFT is mostly employed in quantum physics to solve differential equations, but it may also be utilized to analyze optical-related problems [22]. In recent trends, advanced applications have proposed Fourier transform to preprocess the signal due to the useful characteristics in the time-frequency domain. The representation of the FrlFT signal is illustrated below [23]:

(6) $$FrlFt^{\phi }\left(t,u\right)=F^{\alpha }\left(x\left(t\right)\right)=X_{\phi }\left(u\right)=\int_{-\infty }^{\infty }x\left(t\right)K_{\phi }\left(t,u\right)dt$$

where α  and ϕ=απ/2 represent the order of FrlFT and angle of rotation, respectively. The value of the integer is defined by *n*, while the FrlFT operator defined as $F^{\infty }\left(.\right),$ and kernel function of the FrlFT is computed by using the equation below:

(7) $$k_{\phi }\left(t,u\right)=\left\{\begin{array}{c} \sqrt{\frac{1-jcot\phi }{2\pi }\exp\left(S\frac{t^{2}+u^{2}}{2}cot\phi -Stucsc\phi \right),\phi \neq n\pi }\\ \delta \left(t-u\right),\;for\;\phi =2n\pi \\ \delta \left(t+u\right),\;for\;\phi =2\left(n+\frac{1}{2}\right)\pi \end{array}\right.$$

### 2.2. Signal Filtering

The nature of the ECG signals is non-stationary, which means that the frequency response of the ECG signals varies according to the time. Therefore, the contamination often varies according to the time-dependent variation. Thus, the conventional Fourier-transform technique does not provide the localization of the specific events of the ECG signal, whereas DWT extracts time-based features by removing noisy elements from ECG signals [18]. In order to remove baseline wander and power line interference efficiently from ECG signals, DWT uses two filters, namely low-pass and high-pass, for baseline wandering and power line interference, respectively. After applying these two filters, the central frequency component (CFC) is computed to identify the 0 to 1 wavelet ranges, according to the similarity ratio between the original signal and the wavelet-based chosen component. CFC is identified by using the Daubechies-4 (db4), which is around 0.7. Furthermore, the pseudo frequency (*Fp*) of each scale is computed by using the following equation:

(8) $$F_{p}=\frac{F_{c}F_{s}}{2^{p}}$$

### 2.3. R-Peak Detection

The temporal localization might be lost by performing the Fourier transform on the ECG data. Thus, the purpose of implementing FrlFT is to identify the time-frequency-based features from the ECG signal [24]. FrlFT works as a rotational form when it rotates to the higher value of α, FrlFT extracts the frequency domain, and when it rotates towards the lower value of α, it extracts the time domain values of the ECG signals. To detect accurate high-frequency component, such as R-peak, time localization is the most important and necessary factor, which needs to be considered carefully [18]. In this study, we employed the hit and trial technique for the value of α, and we found that it is equal to 0.0 l; appropriately, this process helps to enhance the R-peaks detection. The dual event-related moving averages technique helps to calculate the ECG cycle by using the following equation:

(9) $$MA_{event}\left(n\right)=\frac{1}{W_{1}}\underset{k=-l}{\sum }x\left(n+k\right),$$

(10) $$MA_{cycle}\left(n\right)=\frac{1}{W_{2}}\underset{k=-p}{\sum }x\left(n+p\right),$$

**Figure 2:**
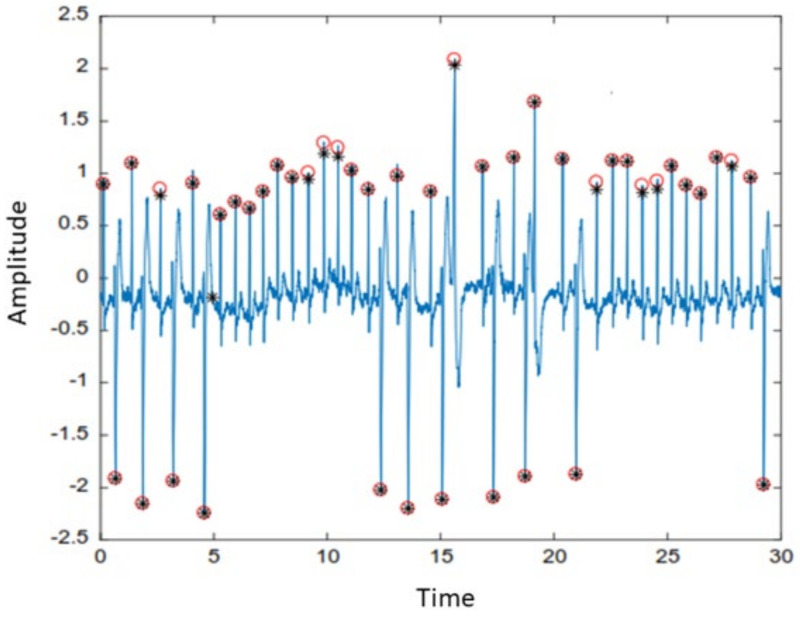
The positive and negative polarity of R−peak detection.

**Figure 3:**
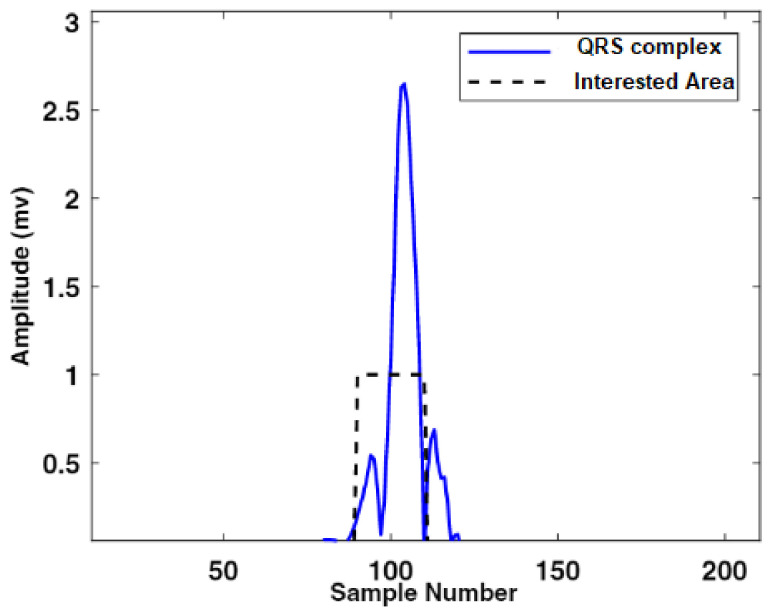
Representation of QRS complex and area of interest.

**Figure 4:**
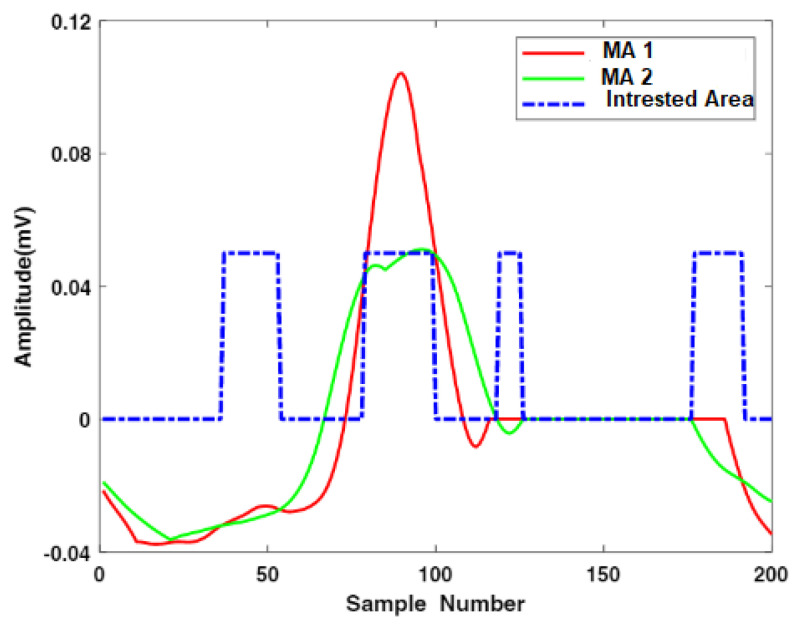
Representation of QRS complex and area of interest using two events.

In this scenario, $W_{1}$ and $W_{2}$ represent the QRS complex and heartbeat durations, respectively. The optimum value of β factor was selected using the hit and trial technique, and it was used to multiply the mean $\left(\mu \right)$ of the enhanced signals. To generate the value of the threshold output number added to the $MA_{cycle}$. The value of the $MA_{event}$ was compared with the associated threshold value. It is stated that if the value of $MA_{event}\left(n\right)$ was more than nth threshold element, 1 is assigned to the new vector; otherwise, it considers 0. The sequence of nonuniform heartbeat is constructed using this method. Moreover, that heartbeat, which has a width equivalent to the $W_{1},$ contains the block of the desired event. The maximum value of the enhanced signal is considered as R-peak in the interesting event block. The accurate detection of the R-peak using the proposed algorithm is shown in Figure 2. Our proposed model also extracts negative polarity of the R-peak, which is beneficial to detect different types of arrhythmias from ECG signals. Figure 3 and Figure 4 represents the area of the interested block and two events results, respectively. To accurately identify the P- and T-peaks in the ECG signal, our proposed algorithm DERMA employs a simplified threshold value, which helps to minimize the computational complexity.

**Figure 5:**
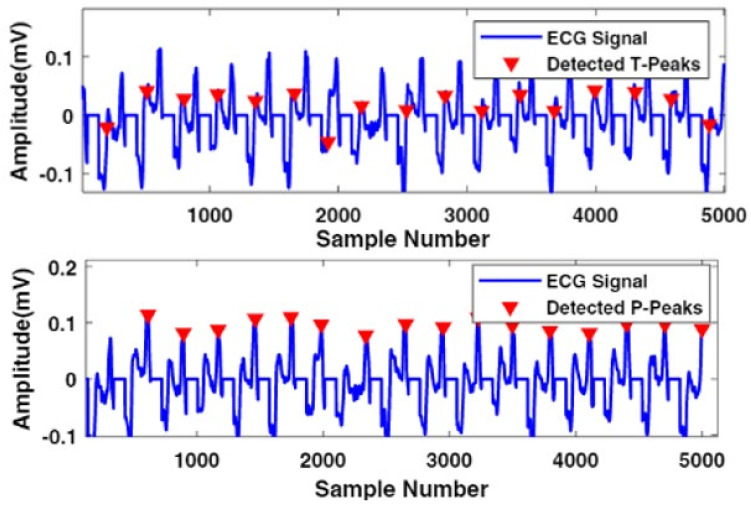
Graphical representation of P and T−peaks detection.

After the elimination process, a total of around 30 samples remained, which are 0.083 s before the eliminated part of the R peak, and 60 samples, which are around 0.166 s after the eliminated part of R; the peak was initialized to 0. However, P and T−peaks occur in the selected samples, and it was almost zero in all types of abnormal heartbeats. After the elimination of the QRS interested block, the FrlFT technique was used for prominent P and T-peaks for the time frequency-based feature extraction. In this scenario, $W_{3}$ and $W_{4}$ represent the peaks and duration of the waves, respectively. Figure 5 represents the extracted P and T-peaks. The following equation defines the DERMA for the calculation of the desired block.

(11) $$MA_{peak}\left(n\right)=\frac{1}{W_{3}}\underset{k=-q}{\sum }x\left(n+q\right),$$

(12) $$MA_{wave}\left(n\right)=\frac{1}{W_{4}}\underset{k=-r}{\sum }x\left(n+r\right),$$

(13) $$q=\frac{W_{3}-1}{2}\;\mathrm{and}\;r=\frac{W_{4}-1}{2}$$

## 3. Classification

Machine learning was used in this section to classify the PVC, LBBB, RBBB, PACE, and APC heartbeats from the ECG signals. In general, machine learning algorithms are trained by using the labeled dataset, where various attributes from each dataset are extracted and formed into a model to predict the outcome. This method is called supervised learning. This method helps to automate the decision-making process by training and testing the different data. However, it includes two major processes, which are feature extraction and classification.

### 3.1. Feature Extraction

This is the unique way to recognize the pattern of diseases from ECG signals using the collection of different types of morphological changes in ECG signals, such as calculation of different peaks, finding intervals, or duration of peaks. These types of features or characteristics distinguish the normal and abnormal events in the ECG signals. Besides this, coefficients of the auto-regressive model help to identify the different characteristics of ECG signals [25]. The order of autoregressive model p, AR (p) is illustrated as:

(14) $$x\left(n\right)=\overset{p}{\underset{i=1}{\sum }}a\left(i\right)x\left(n-i\right)+e\left(n\right)$$

In this scenario, $a\left(i\right)$ is the *i*th coefficient of the AR model, $e\left(n\right)$ is a white noise with a zero mean, and the order of AR model is represented as p. The selection of optimal order is determined by several parameters. The feature-extraction phase is most important because this step determines the better type of input in the ECG signal for classification.

#### Feature Matrix

This is the unique vector that contains the feature-related information of the ECG heartbeats. Two types of datasets are used in this experiment: MIT-BIH arrhythmia [16] and SPNH [26]. Each row contains different types of single ECG heartbeat-related features such as AR order, FrlFT, and interval duration of the peaks, such as QRS complex, P−wave, T−wave, and other temporal values, such as last R-R interval and subsequent R-R interval.

## 4. Supervised Machine Learning Algorithms

ECG signal classification is an important and difficult task. However, it provides detailed information about the abnormality in ECG signals automatically, which provides great benefit to society to reduce the impact of morbidity and mortality around the globe. This proposed model used an SVM and KNN supervised learning algorithm for this experiment. The following subsection briefly describes the machine learning algorithm implementation.

### 4.1. Support Vector Machine Classifier

SVM is under the category of supervised learning technique that mostly solves classification problems. This machine learning algorithm aims to provide the most powerful statistically based learning theory classifier. SVM facilitates the classification of unobservable patterns from the dataset [24]. It is a hyperplane that divides positive and negative sets with maximal margin. The dataset consists based on pairs $\left(a_{1},y_{1}\right),\left(a_{2},y_{2}\right),\ldots \left(a_{N},y_{N}\right),$ where the hyperplane is demarcated as the following quadratic problem:

(15) ∑i=0lαi−12∑i,j=1lαiαjyiyjKXi,Xjα≥0max

(16) $$subject\;to\;\overset{l}{\underset{i=0}{\sum }}\alpha_{i}y_{i}=0$$

(17) $$\alpha_{i}\leq C,i=1,2,\ldots .,l,$$

In this equation $X_{i},\;X_{j}$ represent the input features, whereas $y_{i}$, $y_{j}$ represent the class labels, and $\alpha_{i}\geq 0$ are Lagrangian multipliers, in which C is a constant variable, and K represents the kernel function [27].

### 4.2. K-Nearest Neighbor

KNN has used an instance-based learning method for storing the instances data for training the classifiers using similarity index measures. It is a versatile and robust classifier that is frequently used for more complex classification experiments. KNN essentially determines K instances in the training dataset by using the most popular distance measure technique, Euclidean distance. KNN is one of the simplest and most competitive algorithms, which achieves the highest performance in pattern recognition issues. KNN can give highly competitive results due to its simplistic nature [28].

(18) $$d\left(x_{i},x_{j}\right)=\sqrt{\overset{n}{\underset{r=1}{\sum }}{\left(a_{r}\left(x_{i}\right)-a_{r}\left(x_{j}\right)\right)}^{2}}$$

Whereas the mathematical notation of KNN for *Y* is given as:

(19) $$Y=\frac{1}{K}\underset{x_{i}\in N_{k}\left(x\right)}{\sum }y_{i}$$

where $a_{r}$ and Y shows the value of *r*th variable of an instance *x* and local mean vector, respectively$;\;N_{k}\left(x\right)$ represent as the neighborhood of *x* element [29,30]. In the literature, the KNN classifier is mostly found for classifying abnormalities in the heart, or it is also used to detect heart diseases [20,31,32,33]. Moreover, this machine learning algorithm allows learning the patterns of the dataset from the training feature vector set by using the comparison of the similarity between the test and training feature vector set. Next, we compute the *k*-value using similarity measures that are used to determine the specific class from the dataset.

### 4.3. Evaluation Methods

The current research utilized a 10-fold cross-validation technique for the evaluation and validation of the model. This procedure is repeated until the k times final test result is computed. Moreover, the performance of the DERMA fusion technique is assessed by the various common performance indicators, such as sensitivity (*Se*), specificity (Sp), accuracy (Acc), positive predictivity (PPV), and error rate (er). Four parameters of the confusion matrix are used to derive the performance indicator, namely correctly detected beats (true positive = *TP*), undetected beats (false negatives = *FN*), correctly undetected beats (true negatives = *TN*), and falsely detected beats (false positive = *FP*). These statistical indices are expressed as follows:

Sensitivity measures the average values of positive subjects that are correctly identified from the ventricular arrhythmia in the beat from ECG subjects. It is computed as:

(20) $$Se=\frac{1}{N}\overset{N}{\underset{i=1}{\sum }}\frac{{\left(TP\right)}_{i}}{{\left(TP+FN\right)}_{i}}$$

Accuracy computes the rate of correctly classified ventricular arrhythmia classes out of the total number of ventricular heartbeat subjects. Accuracy is computed as:

(21) $$Acc=\frac{1}{N}\overset{N}{\underset{i=1}{\sum }}\frac{{\left(TP+TN\right)}_{i}}{{\left(TP+FP+TN+FN\right)}_{i}}$$

Positive Predictive Value represents the average value of the subjects with positive ventricular arrhythmia in the heartbeat with truly or correctly classified ventricular arrhythmia. It is defined as:

(22) $$PPV=\frac{1}{N}\overset{N}{\underset{i=1}{\sum }}\frac{{\left(TP\right)}_{i}}{{\left(TP+FP\right)}_{i}}$$

Error Rate is used to calculate the rate of ventricular arrhythmia in the heartbeat using the rate of incorrectly classified arrhythmias out of all the ventricular heartbeat details. It is represented as:

(23) $$Der=\frac{1}{N}\overset{N}{\underset{i=1}{\sum }}\frac{{\left(FP+FN\right)}_{i}}{{\left(TP+TN+FN+ssFP\right)}_{i}}$$

## 5. Results and Discussion 

The performance evaluation is the most important phase to validate model performance for ventricular heartbeat classification.

### 5.1. Simulation Results

In this section, we discuss the results of the two scenarios, including ECG feature extraction and classification of cardiovascular disease.

#### 5.1.1. Feature Extraction Results

DERMA with FrlFT-based fusion technique detects various types of morphological points from ECG signals to efficiently diagnose the disease. The validation of the proposed method was performed using the MIT-BIH Arrhythmia dataset. The main advantage of the proposed algorithm is that it works independently for feature extraction, which means any lead can be selected for the extraction of high-frequency components from ECG signals. Table 1 shows the performance of both datasets, where a total of six types of features are extracted. The performance results here measured the successful detection of the features with true positive and true negatives, and the parameters in Table 1 identify the sensitivity and accuracy, which are observed from the successful detection of the features. Moreover, the detection error rate defines the error that DERMA fails to extract. DERMA fusion resulted in 99.99% sensitivity and accuracy in QRS complex detection and SDSD. Moreover, the extraction of P-wave showed 99.90% sensitivity and 99.99% accuracy. T-wave showed 99.92% sensitivity and 99.98% accuracy. The performance of detecting the previous RR interval and subsequent RR interval is observed at 99.99% and 99.97% sensitivity and 99.97% and 99.98% accuracy, respectively. Furthermore, average error detection is observed at around 0.0015%, which is quite less.

#### 5.1.2. Classification Results

In this study, we trained the classifier using the MIT-BIH Arrhythmia dataset and tested the classifier using the SPNH dataset. Hence, it was observed that around 70% of the feature vector matrix was utilized for the training and 30% utilized for the testing purpose. A total of five types of heartbeat classes were selected to classify in this experiment, namely PVC, LBBB, RBBB, PACE, and APC. Moreover, SPNH contains four types of groups in the dataset, which include supraventricular tachycardia (GSVT), atrial fibrillation (AFIB), sinus bradycardia (SB), and sinus rhythm (SR). As we know, the machine learning algorithm performs better when we increase the quantity of data, and it was noticed that MIT-BIH Arrhythmia has only 48 ECG subjects. Therefore, we used the SPNH dataset, which contains 12 lead ECG signals from 10,646 patients at 10 s long. The main advantage of using this dataset is that it is already denoised, and the database contains 11 common rhythms and 67 different cardiovascular conditions. The parameter for the SVM classifier was set to γ = $\frac{1}{2\sigma^{2}}$ and adjusted to 2.44 × 10^−4^. Hence, it was observed that the performance of the MIT-BIH arrhythmia dataset shows a better performance than the SPNH dataset, which is demonstrated in Table 2. Both datasets have different sampling frequency rates; due to maintaining integrity and simplicity, this study fixed the resampling rate to 128 Hz for both datasets. Therefore, each dataset has a similar type of noisy elements, such as baseline wander and power line interference. Moreover, Table 3 shows the performance comparison of the current study with state-of-the-art methods. According to the Table 3, it was observed that authors in [34,35] achieved almost similar results; however, the limitation of state-of-the-art methods is observed in that these studies only extracted QRS complex from ECG signals, whereas this study extracts all necessary specific events from ECG signals to identify the exact condition of the heart. Only the QRS complex could not provide extensive knowledge about the abnormality in ECG signals. Hence, it is a quite complicated task because we did not found any solid studies in this regard that utilized the SPNH dataset and MIT-BIH Arrhythmia dataset together. Therefore, to fairly compare the algorithm of this study, only the MIT-BIH Arrhythmia dataset was utilized because most of the studies used this dataset in the past for their experiment. In this study, we provide a unique solution to detect the exact condition of the heart by determining five classes of heartbeats, namely PVC, LBBB, RBBB, PACE, and APC. The computational complexity of the proposed model was identified by using three components, including DERMA with FrlFT, and classification models. The computational complexity of our proposed model was calculated around O (N *log*_2_ N), where N represents the number of features. According to the perspective of the computational cost, we observed that our proposed DERMA with fusion technique provided more stability while changes in datasets occurred. Hence, it was also observed throughout the experiment that normalization plays a vital role in both dataset cases.

## 6. Conclusions

DERMA with FrlFT-based fusion technique is thus proposed to extract the features from ECG signals. MIT-BIH Arrhythmia and SPNH datasets were used in this experiment. However, both signals have different sampling frequencies, so in this study, we resampled the ECG signal at 128 Hz to maintain the simplicity and integrity of the algorithm. To preprocess ECG signals, we used the traditional wavelet decomposition method to denoise signals, whereas the usage of the fusion model increased the performance of the detection of morphological values of ECG signals to classify the five classes of heartbeat efficiently. Six types of features are extracted, which identify the actual variation in ECG signals to simplify the detection of abnormal events. Moreover, this study utilized two supervised models for the classification, namely KNN and SVM, which performed overwhelmingly, and we observed that the performance using the MIT-BIH Arrhythmia dataset was more remarkable than the SPNH dataset. Both classifiers were trained and tested using the two datasets, which are quite challenging tasks to integrate; the purpose of using two datasets together was to enhance the size of the dataset to train the learning algorithm efficiently. On the other hand, the reason for using the SPNH dataset was that it contains already preprocessed dataset with unique features. It was observed that our preliminary results are promising and further improved the identification of the different types of abnormalities that can lead to mortality.

## Figures and Tables

**Table 1:** DERMA Fusion performance results of feature extraction using both datasets.

S. No	Feature	Sen %	Acc %	Der %
1	QRS complex	99.99	99.99	0.002
2	P-wave	99.90	99.99	0.001
3	T-wave	99.92	99.98	0.002
4	The previous R-R interval	99.99	99.98	0.001
5	The subsequent R-R interval	99.97	99.98	0.001
6	The standard deviation of successive difference (SDSD)	99.99	99.99	0.002
Total/Average Performance		99.96	99.98	0.0015

**Table 2:** Performance Results of the Classification Models.

Dataset	SVM			KNN		
MIT-BIH	Acc (%)	Sen (%)	PPV (%)	Acc (%)	Sen (%)	PPV (%)
Normal	0.92	0.98	0.98	0.94	0.97	0.92
PVC	1.00	1.00	1.00	0.98	0.95	0.97
PACE	0.95	0.97	0.99	0.99	0.94	0.96
AFIB	0.98	0.99	0.96	0.98	0.93	0.98
APC	0.99	0.98	0.97	0.99	0.96	0.99
**SPNH**						
GSVT	0.70	0.71	0.68	0.59	0.80	0.87
AFIB	0.83	0.82	0.87	0.85	0.88	0.89
SB	0.82	0.84	0.90	0.91	0.92	0.89
SR	0.78	0.79	0.75	0.74	0.79	0.78

Acc, accuracy; Sen, sensitivity; PPV, positive predictivity.

**Table 3:** Performance comparison using MIT-BIH Arrhythmia.

Studies	Acc (%)	Sen (%)	Sp (%)	PPV (%)
[34]	99.88	99.93	NA	99.95
[35]	NA	99.99	NA	99.97
[36]	NA	87	99	85
[37]	92.73	7.35	96.70	88.01
[38]	96.38	97.88	97.56	95.46
[39]	NA	78.8	NA	90.8
**Proposed**	99.98	99.96	99.9	99.98

## Data Availability

The dataset used in this experiment was specifically MIT-BIH Arrhythmia from physionet.org.
